# Successful Strategies and Areas of Improvement–Lessons Learned from Design and Conduction of a Randomized Placebo-Controlled Trial in Palliative Care, ‘Palliative-D’

**DOI:** 10.3390/life11111233

**Published:** 2021-11-15

**Authors:** Maria Helde Frankling, Caritha Klasson, Linda Björkhem-Bergman

**Affiliations:** 1 Department of Neurobiology, Care Sciences and Society (NVS), Division of Clinical Geriatrics, Karolinska Institutet, Blickagången 16, Neo Floor 7, SE-141 83 Huddinge, Sweden; caritha.klasson@ki.se (C.K.); linda.bjorkhem-bergman@ki.se (L.B.-B.); 2 Thoracic Oncology Center, Theme Cancer, Karolinska University Hospital, Solna, SE-171 64 Stockholm, Sweden; 3 Stockholms Sjukhem, Palliative Medicine, Mariebergsgatan 22, SE-112 19 Stockholm, Sweden

**Keywords:** randomized controlled trial, placebo-controlled, palliative care, palliative care research, research, clinical trial, recruitment, survival prediction

## Abstract

Clinical trials in palliative care are challenging to design and conduct. Burden on patients should be minimized, while gatekeeping by professionals and next-of kin needs to be avoided. Clinical deterioration due to disease progression affects attrition unrelated to intervention, and different care settings complicate comparisons and reduce the generalizability of the results. The aim of this review is to provide advice for colleagues planning to perform clinical trials in palliative care based on our own experiences from performing the Palliative-D study and by a thorough literature review on this topic. The Palliative-D study was a double-blind trial with 244 randomized patients comparing the effect of vitamin D_3_ to placebo in patients with advanced or metastatic cancer in the palliative phase of their disease trajectory who were enrolled in specialized palliative home care teams. Endpoints were opioid and antibiotic use, fatigue, and QoL. Recruitment was successful, but attrition rates were higher than expected, and we did not reach targeted power. For the 150 patients who completed the study, the completeness of the data was exceptionally high. Rather than patient reported pain, we choose the difference in the mean change in opioid dose between groups after twelve weeks compared to baseline as the primary endpoint. In this paper we discuss challenges in palliative care research based on lessons learned from the “Palliative-D” trial regarding successful strategies as well as areas for improvement.

## 1. Introduction

The design of clinical studies involving patients with a limited remaining life span requires careful planning. Patients with metastatic or locally advanced cancer often have a severe symptom burden encompassing physical, social, and existential dimensions. They are often heavily medicated, and further pharmacological intervention increases the risk of side effects and drug–drug interactions. Trials involving patients in a palliative setting should thus aim at minimizing the side effects of intervention, discomfort from procedures, and negative psychological reactions. Fluctuation in the symptom burden over time, individualized pharmacological interventions, comorbidities, and progressive illness are challenges facing the researcher planning such trials [1]. In addition, ethical considerations regarding the beneficial perspective for the patient to participate in a placebo-controlled trial when facing a life shortening disease need to be made [2]. The aim of this review is to provide good advice for colleagues planning to perform clinical trials in palliative care based on our own experiences from performing the Palliative-D study and by a thorough literature review on this topic.

On an organizational level, resources and gatekeeping by professionals and heterogenous cohorts in palliative care facilities need be addressed [1,3,4,5]. Still, it is important to gather evidence on pharmacological symptom management in heterogenous palliative care cohorts, preferably through the use of RCT:s since we often still lack a sound evidence base for pharmacological interventions regarding, for example, fatigue and breathlessness [6,7].

A systematic review on reporting in palliative care trials identified pharmacological studies on symptom control regarding physical dimensions in patients with cancer as the most common types of trial [8]. Four out of ten trials lacked a defined primary outcome, and 137 different scales and questionnaires were used to assess outcome [8]. A total of 2% of studies were evaluated as having a low risk of bias according to the Cochrane Risk of Bias tool, and slightly more than one third of studies reached their inclusion goals [8]. During a review of the scientific literature on palliative care from 2004 and 2009, only 6% of the trials were RCTs [9]. In 2014, Aoun concluded that Cochrane systematic reviews of palliative care interventions have difficulties at arriving at firm conclusions due to methodological problems [1]. This problem persists in more recent Cochrane reviews on pharmacological interventions in palliative care settings [6,7,10,11].

“Palliative-D” was a double-blind placebo-controlled randomized trial (1:1) testing the hypothesis that the correction of vitamin D deficiency could reduce opioid use in cancer patients who have been admitted to palliative care [12]. The effects on antibiotic use, vitamin D levels, fatigue, and quality of life (QoL) were also assessed. The hypothesis that vitamin D supplementation to vitamin D deficient patients could reduce pain and infections and possibly reduce fatigue and improve QoL was based on both mechanistic and clinical data. The induction of the synthesis of antimicrobial peptides [13,14] as well as a dampened inflammatory response [15,16] are possible mechanisms supporting a clinical effect of vitamin D regarding infections and pain. Further, positive results from a non-randomized study regarding vitamin D supplementation in patients with advanced cancer warranted the conduction of a RCT [17].

Patients were recruited from three home-based palliative care facilities in Stockholm, and according to study protocol, patients with advanced or metastatic cancer (any type of cancer) with a physician-predicted estimated survival time of more than three months and a vitamin D deficiency could be enrolled. The intervention period was twelve weeks, and the outcome measures were assessed at baseline and monthly thereafter. A small, dedicated study team with part-time nurses and physicians reviewed patient lists for eligible patients, screened, and randomized all of the patients. We used results from a cross-sectional study showing an association between vitamin D and opioid dose in patients with cancer in the palliative phase of their disease trajectory [18] as well as a pilot study with vitamin D supplementation to help us attain funding for the RCT, for power calculation, and for the statistical analysis plan [19]. A detailed study protocol was published previously [17], as has the demographic data of the screening cohort [20].

**Figure 1:**
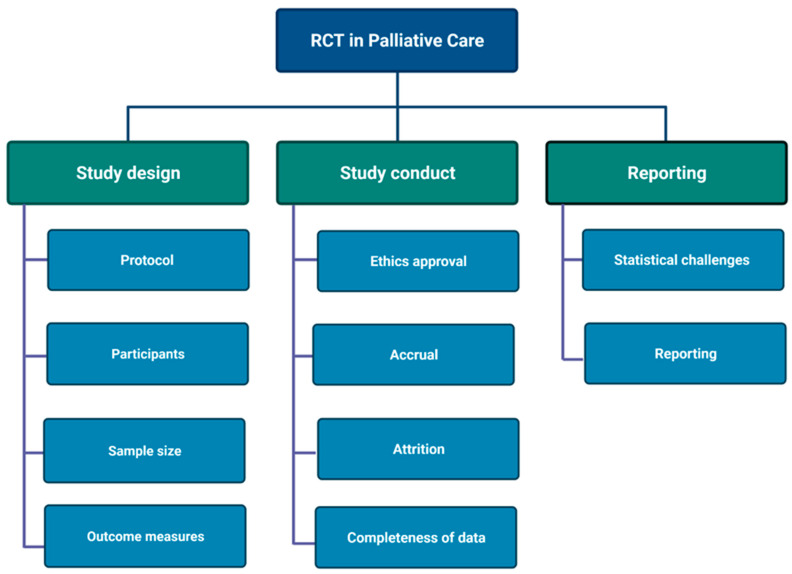
Important elements to consider when designing a RCT in palliative care. Made with Biorender.com (accessed on 9 November 2021).

The research methodology regarding intervention studies in palliative care have previously been discussed in systematic methodological reviews [8,9,21,22,23], narrative reviews by collaborations of palliative care researchers suggesting frameworks and check-lists to optimize future trials [1,3,24,25,26,27], surveys and qualitative of analyses of the attitudes of patients and professionals [4,28,29], lessons learned from individual trials [30,31,32,33,34,35], and suggestions for new types of trial designs [36]. The following methodological discussion on the design, conduction, and reporting of the “Palliative-D” trial is structured to emphasize successful strategies as well as less wise choices that were made during the trial process. We hope that our experiences will help other clinical trialists in palliative care when planning future research projects. In Figure 1, we present an outline of important elements to consider when designing a RCT in palliative care.

## 2. Methods

### 2.1. Study Design

#### 2.1.1. Protocol

Based on earlier and ongoing palliative care trials, the Australian Palliative Care Clinical Studies Collaborative (PaCCSC) identified minimal burden on participants and clinical staff regarding questionnaires and diaries and the use of routinely collected data as being crucial to successful trial completion [27]. In “Palliative-D”, we adhered to this by using data routinely collected outcome data from ESAS, which were entered biweekly during the trial into the electronic medical records during nurse visits to the patients’ homes. We also used outcome measures (opioid dose, antibiotic use), from which data could be retrieved from medical records by the research team without engaging regular staff.

Our study protocol allowed for flexibility regarding follow up since all visits could be scheduled within a timeframe of +/− seven days from the pre-planned date [17]. We could thus use regular visits by team nurses that were sometimes rescheduled due to both patient-related as well as organizational reasons to hand out the study drug, draw blood samples, and to collect the patient-reported outcome data. The flexibility regarding follow-up appointments ensured the collection of data from nearly all time points in all of the patients who completed the study. Since the effects of vitamin D supplementation develop over time, we also designed a trial with a three-month long intervention. This was conducted in contrast to recommendations for trials in palliative care to cover the shortest possible intervention time frame to avoid high attrition rates due to patient deterioration [3,24,27].

In our protocol, we changed one inclusion criterion from the pilot study by lowering the threshold 25-hydroxyvitamin D (25-OHD) level from 75 to 50 nmol/L since patients with lower 25-OHD levels profit more from vitamin D supplementation [37]. In both the observational and investigational study from our team preceding the RCT, mean serum levels of 25-OHD were well below 50 nmol/L [18,19]. We did not conduct a detailed assessment on eligible patients, as suggested by Hagen et al. when planning the RCT [38]. Instead, we took for granted that the change in the inclusion criteria would not compromise the inclusion rates based on our previous results. However, the patients who were screened for the RCT had less advanced disease than the patients in the previous studies, and the median 25-OHD in the screened cohort was instead 51 nmol/L [20]. We thus had to screen more than twice the number of patients who were finally randomized in the RCT, prolonging the trial and requiring more resources overall [12,20].

#### 2.1.2. Participants

Previous experiences from palliative care trials advocate broad inclusion criteria to mirror the heterogeneity of the target population and to facilitate patient recruitment [27] [23]. During our recruitment process, we were already able to see that we had to exclude many otherwise eligible patients who were prescribed 400 IE of daily vitamin D in combination with calcium since any type of ongoing vitamin D supplementation was an exclusion criterion. To solve this problem, we changed the inclusion criteria to allow for low dose vitamin D supplementation that would not affect outcome measures, ensuring sufficient recruitment rates [12,17]. This change was made through a protocol amendment two months after the start of the trial [12].

#### 2.1.3. Sample Size

In palliative care RCTs, it is recommended to inflate the sample size to allow for attrition rates of 25–40% [26,27]. In earlier palliative RCTs in palliative care, sample size calculations were only reported in one third of trials [39], while a more recent review found that more than half of RCTs reported this [8]. We based our power calculation on the pilot study and assumed an attrition rate of 25% [17,19]. This assumption proved to be too conservative since 39% of randomized patients did not reach end-of-study [12].

#### 2.1.4. Outcome Measures

In palliative care, patient-reported outcomes using evidence-based instruments is the golden standard for the evaluation of interventions, both in clinical trials and increasingly in routine clinical care. In the past decade, initiatives to increase, improve, and standardize the use of outcome measures in palliative settings [40,41] have resulted in guidelines such as the EAPC White Paper [42]. Yet, there is still a large amount of diversity in terms of the use of questionnaires and assessment instruments [8].

In “Palliative-D”, we used outcome data from the well-validated ESAS-scale [43,44,45], which, as mentioned above, were regularly collected in routine care at our facility and were entered directly into patients’ electronic medical records during the nurse visits to the patients’ homes. With the aim of capturing broader constructs, we added assessment with EORTCQLQ-C15-PAL [46,47,48]. Since EORTC-QLQ-C15-PAL is not integrated into our medical record system, paper questionnaires were sent out to patients and were then transferred back to the study team after completion. To avoid an unduly burden on regular team nurses, we limited the use of EORTC-QLQ-C15-PAL to screening and to the last visit after 12 weeks, which prevented us from capturing fluctuations in reported outcomes over time [12,17]. There were also discrepancies in the results regarding fatigue and QoL in our cohort when using the different instruments, complicating the interpretation of the results [12,20]. Still, using two different instruments gave us the opportunity to form a post hoc analysis of the outcomes used in both instruments. In addition, an opportunity to further validate the instruments for both clinical practice and in research were presented by performing a psychometric analysis for Swedish conditions in palliative care.

The use and doses of pharmacological agents are not regularly used as outcome measures in palliative care, and only rarely has opioid dose been used as a proxy of pain in other settings [49]. In the case of “Palliative-D”, we chose the difference in the change in the opioid dose after twelve weeks as the primary endpoint, which is in line with findings from the association study and pilot study [18,19]. Since the pilot study rendered a significant difference regarding opioid use between vitamin D-treated patients and matched controls irrespective of baseline opioid dose (including zero opioid use at time of randomization), we refrained from adding ongoing opioid medication to the list of inclusion criteria in the subsequent RCT [17]. However, as discussed in the original publication, the patient cohort randomized in “Palliative-D” were earlier in their disease trajectory, and a large group of patients did not need long-lasting opioids at any time-point during intervention [12]. This diluted the results and weakened the reported association between vitamin D supplementation and opioid use [12]. The inconsistency between the studied cohorts in the pilot study and the RCT highlights the fact that even the use of pilot studies to plan larger trials does not ensure entirely successful study protocols [50,51].

The outcome of antibiotic use as proxy for infections in vitamin D research has been used in other settings, and infectious burden cannot be assessed using patient reported outcome measures [52,53,54,55]. In the present RCT, data collection and interpretation regarding this outcome was unproblematic [12].

#### 2.1.5. Data Collected at Screening

In “Palliative D”, all of the patients who consented to participating in the trial had their individual vitamin D levels measured as part of the screening assessments. Patients with levels of 25-OHD > 50 nmol/L (more than 50% of the screened population) were not randomized to the study drug [12,20]. We thus ended up with a large screening cohort (n = 530), with comprehensive data on demographic variables, baseline outcome measures, and survival [20]. This allowed for descriptive and association studies in a larger cohort. A study on gender differences regarding fatigue has already been published [20]. Further, blood samples for biobanking were collected at screening and end-of study, permitting future translational research in a palliative care cohort [17].

To ensure the external validity of the results and to enable comparisons, a thorough demographic description of palliative patient cohorts regarding socioeconomic indices, type of life-limiting illness, phase of illness, and physical performance status at time is of importance [23,25,56]. Still, reporting on these variables leaves room for improvement [23,56]. In “Palliative-D”, we lacked both baseline data on socioeconomic variables and baseline physical status since this information was not routinely collected in our palliative care facility.

#### 2.1.6. Name of Trial

Use of the word “palliative” in the name of the trial was uncontroversial as far as the Ethics committee was concerned. However, a few patients and next-of-kin reacted negatively to receiving information on a trial called “Palliative-D” since they had not perceived that the illness had reached a palliative stage. This is in line with qualitative approaches to evaluating the conduction of RCTs in palliative care, where it was acknowledged that patients and family caregivers were not always fully aware of the patient’s condition and the transition to palliative care [28]. The concept of palliative care itself may be challenging for patients and relatives to grasp [57] and is generally regarded by the public of being representative of the last days of life [58]. Further, the introduction of new oncological treatment modalities and differences between how oncology specialists, palliative care professionals, and patients use terminology related to disease phase may lead to misperceptions [59,60,61,62]. In this study, this yielded an ethical dilemma, as revealing information concerning estimated lifespan could affect the patient negatively. Actions to minimize the risk were taken by consulting the team at an early stage to avoid approaching patients who had not accepted that they were in fact in a palliative stage of their disease trajectory.

## 3. Results

### 3.1. Study Conduct

#### 3.1.1. Ethics Approval and Application to Medical Products Agency

We started the preparing study protocol, patient information, and the application to the ethics committee and the Swedish Medical Products Agency more than one year before the start of the trial. The feedback from regulatory bodies was constructive and helped us improve our protocol. Still, it is important to take into account that preparations for a clinical trial are time consuming and that sufficient resources need to be allocated for the process to move on [35]. To improve recruitment rates, we adjusted the exclusion criteria as described above and also extended the trial from one to three sites, necessitating amendments to the study protocol [12]. Further, we obtained acceptance from the Ethics committee and from the Medical Products Agency to exclude “death” as a serious adverse event and to only record previously described side effects of vitamin D_3_ as adverse events. This facilitated study conduction considerably.

#### 3.1.2. Accrual

Patient accrual can be challenging due to gatekeeping by both clinicians and next-of-kin [28,63,64]. In a qualitative study on Swedish palliative home care facilities, the communication of the RCT-design to patients and family caregivers and the contradiction between the regular palliative care approach of offering patients all possible support as well as the withholding of intervention in the comparator group in a randomized trial were identified as obstacles in the recruitment process [28]. Still, several studies show that patients are willing to participate in clinical trials, even in late palliative stages [65,66]. A recently published survey from Japan identified quick and easy trials with oral medication without side effects as important for patient participation, while the RCT design was associated with an unwillingness to participate [29].

In “Palliative-D”, one out of the three study sites had a research facility. In a second site, one researcher had conducted smaller studies previously. In most cases, the staff was not experienced in recruiting and retaining patients in clinical trials. Acknowledging this, we tried to reduce gatekeeping by staff through information meetings, regular newsletters during the first months of the trial, and a flexible approach by the study team to minimize the extra workload. At the start of the trial, the study physicians screened all of the admitted patients for eligibility in cooperation with the patient’s responsible physician when needed to address gate-keeping issues. Once the trial was up and running, we planned to let each palliative care team identify newly referred patients who were eligible for the study and to pass this information on to the study team. This did not work out, however, and to ensure continuous patient accrual, the study physicians instead continuously reviewed patient lists to identify newly referred patients. We think that both workload and unfamiliarity with clinical research were obstacles in engaging regular staff in patient accrual. In a qualitative study, the research group will use staff informants to gain more insight into this matter.

Soon after starting the trial, we noticed that the patients who had been recently referred to palliative care often declined receiving oral and/or written information about “Palliative-D”. They informed us that this was because they already had a great deal of new information to absorb and that they had frequent visits by the multi-professional team. In these cases, a successful strategy was to schedule a new contact a few weeks later, when the patients had adjusted to their new routines. Patients whose lab results or current medication did not meet the inclusion criteria at time of the referral to the palliative care team were screened for eligibility once a month for as long as they were admitted to the palliative care team. Thus, we could recruit patients whose hypercalcemia or renal failure resolved with treatment over time as well as patients who stopped taking medications excluding them from participation, such thiazides or digitoxin.

Thus, we could recruit patients whose exclusion criteria disappeared over time, such as hypercalcemia or renal failure resolving with treatment as well as patients who stopped taking medications excluding them from participation.

We also offered all of the patients who were randomized in the trial a bottle of vitamin D_3_ oil drops after the completion of trial activities and informed the patients about this during the screening process.

#### 3.1.3. Attrition

In a UK study on publicly funded RCTs, the proportion of recruited patients with valid outcome data at follow-up was 89%, and nearly four in five trials reached the target sample size [67]. In contrast, RCTs in palliative care have higher attrition rates, with a recent review estimating the total attrition to be 29% [68]. The high attrition rate is mainly attributed to deterioration due to underlying illness or to death. Prognosticating remaining lifespan in patients with advanced cancer remains a challenge, where medical professionals tend to overestimate survival time [69,70]. To this end, several prognostic instruments for use in palliative care populations have been developed. However, they are not yet widely used, and discussion regarding best practice in this field [8,22,71,72,73,74] as well as research on algorithms based on machine learning continues [75]. As mentioned above, we had relatively high attrition rates in “Palliative-D” and would have possibly been aided by a standardized instrument to help us prognosticate the patients’ risks of quick deterioration.

#### 3.1.4. Completeness of Data

In contrast, the retention rates for patients who fulfilled all of the follow-up visits and procedures in “Palliative-D” were exceptionally high, with very little missing data [12]. We have identified flexibility regarding study visits addressed above as a successful strategy. Further, the presence of a small, dedicated study team, whose members discussed practical issues regarding trial patients on an almost daily basis and who were involved in regular care at two of the three sites facilitated data collection. Additionally, the limited geographical area from which patients were recruited made it possible for us to deliver study drug to patients, hand out or pick up left-behind questionnaires, or draw extra blood samples, if needed. Both involvement in clinical work and geographical proximity were stressed as successful strategies in other trials [30]. The study team members had access to portable computers with both electronic charts and an electronic database, which facilitated data handling. The same electronic medical record was used in all three study sites, reducing the obstacles facing many other palliative care researchers [76].

### 3.2. Reporting

#### 3.2.1. Statistical Challenges

IN RCTs, the intention to treat-analysis (ITT) is performed on all randomized patients. Statistical imputation techniques compensate for missing data from patients who no longer participate in the trial at follow-up. Imputation techniques, however, focus on data that are missing “at random”, which is not always the case in palliative care RCT:s [68]; therefore, choosing a well-suited method might pose a challenge [26]. Improper imputation techniques and performing imputations in small data sets may introduce new bias [77], and it has been suggested that a pattern of missing data should be investigated to choose the imputation method in palliative care trials [26]. It has been argued that palliative care trial populations should be treated differently from other types of cohorts since attrition is largely due to clinical deterioration or death. An ITT-analysis then reduces power and creates a systematic bias away from the true effect [77].

In a “per protocol” analysis, only patients who complete the trial and from whom data has been collected on all pre-specified data collection points are included. This approach increases the risk of reducing differences between groups, and on missing information on adverse events.

To help palliative care researchers analyse their often incomplete data sets, Currow et al. propose a modified palliative-ITT analysis approach “on a continuum between ITT and per protocol analysis”, where data from deceased patients should not be included in the analysis of the primary outcome given that it is prespecified in the original protocol and because deterioration is undebatable due to disease progression; an independent data Monitoring Committee also identifies deterioration as such, and dropout is shown in a CONSORT flowchart [77]. In addition, sensitivity analyses should help identify attrition that is not at random. This approach is however not generally accepted, as seen in a recent debate [78,79,80].

In “Palliative-D”, we presented both an ITT-analysis without imputed data from patients who were excluded “not at random” and a per protocol analysis [12]. In addition, we also presented and an ITT-analysis with imputed data in Supplementary files.

#### 3.2.2. Reporting

Reporting on palliative care trials face the difficulties of describing heterogenous cohorts and diverse care setting, and these challenges are aggravated by a lack of standard definitions [1,4,8,25,27,81,82,83]. In reporting the results of “Palliative-D” we adhered to the CONSORT guidelines [84] and also tried to adhere to the “framework for generalizability” proposed by Currow et al. [25]. Still, we lacked data on some variables such as socioeconomic factors. Due to limited word counts in original articles reporting RCTs, information on the organization of the Swedish health care system and of our palliative care facilities is provided in the Supplementary Materials in the original article [12]. Although we tried to be as stringent and detailed as possible in our description of the general setting in which the trial was conducted, the review process for the original article made us aware that these matters are challenging to present in a clear way.

Further, the detailed reporting of reasons for the non-completion of the 12-week intervention period in the “Palliative-D” study are presented in the original publication [12]. However, a review of these cases by an independent monitoring committee would have added to credibility of results.

In Table 1, we present important aspects of the study design and study conduction regarding “Palliative-D” in relation to previously proposed best practice.

## 4. Discussion

Any clinical researcher in every discipline of medicine sets out to plan and conduct trials that are feasible and that can reach a precise and unbiased answer to the targeted research question. Good intentions set aside, these researchers more often than not need to discuss the limitations in their material that compromise the possibility of establishing causal relationships or applying present findings to other clinical settings. As seen in the Results section, “Palliative-D” turned out to be a well-conducted trial, but with high attrition rates that rendered the trial under-powered, inclusion criteria that were so wide that they diluted the treatment effect, and primary endpoints that are not unanimously accepted in palliative care research [12]. Previous studies highlight several barriers, both organizational and in the decision-making process, for trials conducted outside of the hospital setting [85]. The suboptimal design and conduction of trials in palliative care is especially troublesome since few patients will live long enough to profit from the possible new clinical guidelines. In addition, these patients already have to cope with severe symptom burdens and the awareness of a limited lifespan. Further, they are often heavily medicated, and further pharmacological intervention increases the risk of side effects and drug–drug interactions. Mutual trust between the physician and the patient have been stated as a promoting factor for trials conducted in the palliative care context to address these ethical issues [86].

Another challenge when conducting research in a palliative care is the heterogeneous patient group. Including patients that reflect the true clinical situation in the palliative care setting in randomized clinical trials, i.e., a heterogeneous population of different cancer types and different ongoing treatments, as was the case in the Palliative-D study, will make the results more valid to the clinical setting and thus more generalizable. On the other hand, strict inclusion criteria regarding cancer type and ongoing treatment will result in a more homogenous study cohort, making it easier to answer a specific research question. However, the results will then only be valid for a specific type of patient and not for palliative care patients in general.

During the process of analysing and reporting results from “Palliative-D”, we reflected on the strengths and weaknesses regarding the conduction of clinical trials in our specific geographical and organizational setting. We identified that, in addition to matters already addressed in the results section, sufficient funding and staff allocation were crucial to trial success. Further, collaboration with other palliative care researchers might have improved the protocol.

In “Palliative-D”, the principal investigator applied for and received funds for planning and starting the trial, and when recruitment rates were promising, the principal investigator was ensured financial funds to also complete the trial. The possibility of taking time off from clinical work both when planning the trial and when writing the protocols, and most notably, during the first weeks of recruitment, were crucial to a successful study start. In a non-academic clinical setting, a dedicated and agile study team that could screen patients for eligibility, plan all of the data collection events, follow up with patients regarding adverse events and compliance as well as aid regular staff with study related procedures when needed was essential for recruitment and retention.

Crucial for accrual and retention was a six-week long start-up period, during which a small team comprising one study physician and one study nurse worked in close collaboration. They contacted and assessed all of the eligible patients that were already admitted to the largest participating palliative care facility, thereby giving recruitment a head start. Practical details regarding patient recruitment, randomization, documentation, and planning of follow up visits were continuously adjusted to ensure that resources were used wisely. Checklists for all of the steps in the process were created to ensure high quality data collection and documentation. Dialogue with regular palliative care team staff helped us to understand how the processes could be improved and ensured a positive attitude towards the trial in the involved palliative care teams. To conclude, the grander scale of a large RCT showed the need for more detailed planning and conduction of everyday study procedures compared to the pilot study, and sufficient resources were crucial for success.

Collaborations of palliative care researchers and networks for clinical trialists have been a key to success in different parts of the world [24,30,87,88]. Swedish palliative care research has the advantage of strong and successful collaborations regarding registry-based research [89,90] as well as research on outcome instruments, qualitative studies, and implementation of palliative care methods [91]. However, experiences from planning and conducting randomized intervention trials are scarce. We believe that discussions with wider range of experienced clinical researchers may have improved our protocol, and that in a smaller country such as ours, collaboration groups involving both palliative care researchers and researchers from other disciplines might be necessary.

## 5. Conclusions

If we were to conduct the study again, we would narrow down the inclusion criteria; use a validated physical assessment scale; collect data on socioeconomic variables as part of the screening procedure; create a collaboration group for ideas, input, and strategic discussion; and use an independent monitoring committee. We would once again use a small, focused group of investigators involved in clinical work at the site and conduct the trial in a setting with geographical proximity. Further, we would stick to the concept of using endpoints on which data can be easily collected without participation from the study subjects or staff, such as antibiotic and opioid use. These endpoints could be assessed in all patients and ensured enough data for statistical analysis even though fewer than expected patients could complete the trial.

## Figures and Tables

**Table 1:** Aspects on study design and conduction in relation to previously proposed best practice.

Aspect of Study Design/Conduct	Proposed Success Factors/Best Practice	Practical Application in “Palliative-D”	Comment
Protocol	Minimize burden on participants and clinical staff [27]	Outcome data retrieved from medical records Visits could be rescheduled +/−7 days	Very little missing data
	Align protocol with standard clinical practice [27]	Routinely collected outcome data (ESAS), outcome data collected at regular nurse visits	Very little missing data
	Establish and monitor key performance indicators for recruitment and screening [27]	The study group continuously monitored this and changed study protocol to include more sites and to widen inclusion criteria	Improved accrual
	Collect detailed demographic data to ensure generalizability [25]	We did not collect socioeconomic data	Reduces generalizability
	Standardized assessment of physical performance status [25]	We did not use a validated instrument	Reduces generalizability
Participants	Keep inclusion and exclusion criteria as broad as possible [27]	Early change in inclusion criteria to allow for daily small intake of vitamin D	Improved accrual
	Ensure eligibility criteria can be applied uniformly across sites [27]	All sites in one region, uniform health care system	Ensured accrual
Sample size	Allow for attrition rates of 25–40% [27]	Sample size calculation on 25% attrition rate	Higher than expected attrition rate
Outcome measurement	Assess primary endpoint data to occur as soon as clinical benefit is likely to occur [27]	3-month follow-up due to slow onset of effects of vitamin D	High attrition rate due to deterioration and death
Analysis plan	Modified ITT [77]	In our analysis we prespecified both ITT and PP analysis	We discussed using modified ITT with reviewers, but ended up with ITT with imputation and PP
Study conduct	Provide support and coordination from a central office [27]	Karolinska Trial Alliance were contracted for help with initial study protocol and biobank	Valuable when working in a small team without administrative resources
	Promote routine screening of inclusion criteria [27]	We initially promoted screening of patients at time of enrolment in our teams	This did not work out; instead, a dedicated study team worked on accrual and retention
	Convince clinicians of the importance of research for improving quality of care, regardless of results, maintain regular communication between sites [27]	Meetings before study start, regular newsletter at beginning of trial, regular team visits by study nurse, reporting of results after data analysis	Many team-members engaged in study procedures, positive attitude in all teams.

## Data Availability

Raw data from the “Palliative-D” study is available from the corresponding author upon request.
